# Impacts of climate change and environmental degradation on children in Malaysia

**DOI:** 10.3389/fpubh.2022.909779

**Published:** 2022-10-14

**Authors:** Mazrura Sahani, Hidayatulfathi Othman, Soo Chen Kwan, Liew Juneng, Mohd Faiz Ibrahim, Rozita Hod, Zul'Izzat Ikhwan Zaini, Maizatun Mustafa, Issmail Nnafie, Lai Che Ching, Ramzah Dambul, Helena Varkkey, Vera Ling Hui Phung, Siti Nur Hanis Mamood, Norhafizah Karim, Nur Faizah Abu Bakar, Muhammad Ikram A. Wahab, Siti Shahara Zulfakar, Yanti Rosli

**Affiliations:** ^1^Center for Toxicology and Health Risk Studies (CORE), Faculty of Health Sciences, Universiti Kebangsaan Malaysia, Kuala Lumpur, Malaysia; ^2^Centre for Earth Sciences and Environment, Faculty of Science and Technology, Universiti Kebangsaan Malaysia, Selangor, Malaysia; ^3^Department of Community Health, Faculty of Medicine, Universiti Kebangsaan Malaysia, Kuala Lumpur, Malaysia; ^4^Faculty of Health Sciences, Universiti Teknologi Mara, Penang Branch, Pulau Pinang, Malaysia; ^5^Legal Practice Department, Ahmad Ibrahim Kulliyyah of Laws, International Islamic University, Kuala Lumpur, Malaysia; ^6^Climate and Environment, UNICEF Malaysia, Putrajaya, Malaysia; ^7^Faculty of Humanities, Arts and Heritage, Universiti Malaysia Sabah, Kota Kinabalu, Malaysia; ^8^Department of International and Strategic Studies, Universiti Malaya, Kuala Lumpur, Malaysia; ^9^Center for Climate Change Adaptation, National Institute for Environmental Studies (NIES), Tsukuba, Japan; ^10^Center for Diagnostic Therapeautic and Investigative Studies (CODTIS), Faculty of Health Sciences, Universiti Kebangsaan Malaysia, Kuala Lumpur, Malaysia

**Keywords:** environmental degradation, climate change, children, marginalized communities, Malaysia

## Abstract

The impacts of climate change and degradation are increasingly felt in Malaysia. While everyone is vulnerable to these impacts, the health and wellbeing of children are disproportionately affected. We carried out a study composed of two major components. The first component is an environmental epidemiology study comprised of three sub-studies: (i) a global climate model (GCM) simulating specific health-sector climate indices; (ii) a time-series study to estimate the risk of childhood respiratory disease attributable to ambient air pollution; and (iii) a case-crossover study to identify the association between haze and under-five mortality in Malaysia. The GCM found that Malaysia has been experiencing increasing rainfall intensity over the years, leading to increased incidences of other weather-related events. The time-series study revealed that air quality has worsened, while air pollution and haze have been linked to an increased risk of hospitalization for respiratory diseases among children. Although no clear association between haze and under-five mortality was found in the case-crossover study, the lag patterns suggested that health effects could be more acute if haze occurred over a longer duration and at a higher intensity. The second component consists of three community surveys on marginalized children conducted (i) among the island community of Pulau Gaya, Sabah; (ii) among the indigenous Temiar tribe in Pos Kuala Mu, Perak; and (iii) among an urban poor community (B40) in PPR Sg. Bonus, Kuala Lumpur. The community surveys are cross-sectional studies employing a socio-ecological approach using a standardized questionnaire. The community surveys revealed how children adapt to climate change and environmental degradation. An integrated model was established that consolidates our overall research processes and demonstrates the crucial interconnections between environmental challenges exacerbated by climate change. It is recommended that Malaysian schools adopt a climate-smart approach to education to instill awareness of the impending climate change and its cascading impact on children's health from early school age.

## Introduction

The United Nations High Commissioner for Refugees (UNHCR) defines a child as “a human being below 18 years under the law applicable to that child” (1). There are currently 2.3 billion people below 18 years old globally (2), accounting for ~30% of the world population (3). In Malaysia, 9.1 million of the total population are children under the age of 18 (4). Climate change is altering the earth's systems in many ways that threaten children's physical and mental wellbeing (5). The global average temperature is already 1°C above pre-industrial times; hence, the likelihood of current children experiencing a world that is on average 1.5, 2 or 3°C above pre-industrial times is significantly higher than that of adults. Climate change poses a significant threat to children's health because children have unique metabolism, behavior, physiology, cognitive and developmental characteristics compared to adults (6, 7).

In Malaysia, rising sea levels and temperatures are causing more floods, less food and water shortages. Additionally, droughts and floods have been linked to increased water pollution and pesticides in food (8). Ambient air quality is sensitive to the effects of climate change and the weather (9, 10). In Malaysia, haze events due to forest and peatland fires have been common, especially during years of intensified dry weather (i.e., induced by the El-Niño phenomenon) (11). These forest and peatland fires (hereafter referred to as wildfires) have contributed to haze events and poor air quality in Malaysia. Climate scientists have predicted that, by 2030, about a quarter of Malaysia's population will be displaced because of climate change.

Despite the many ways climate change impacts children, they are consistently overlooked in the design and content of climate policies and related processes. As UNICEF noted, only 42% of countries' Paris Agreement climate action plans mentioned children or youth (12). This could be due to inadequate evidence guiding policies and plans on the impacts of climate change, as well as environmental pollution and degradation on children's healthy growth, development and socialization. In addition, insufficient attention has been directed to increasing community understanding of the impacts of climate change and environmental degradation on the lives of families and children—particularly those in vulnerable communities—and encouraging environment-friendly values and practices among children and young people. In marginalized communities, climate- and environment-related risks are further exacerbated by poverty, illiteracy and limited access to information. The marginalized groups here include persons with disabilities, youth, women, members of minority groups, indigenous people, internally displaced persons and non-nationals (13). In addition, urban poor communities (based on the country's poverty line income—B40) can also be counted among these marginalized communities, as low family income can increase the impact of climate- and environment-related risks, such as food insecurity and internal displacement.

**Figure 1:**
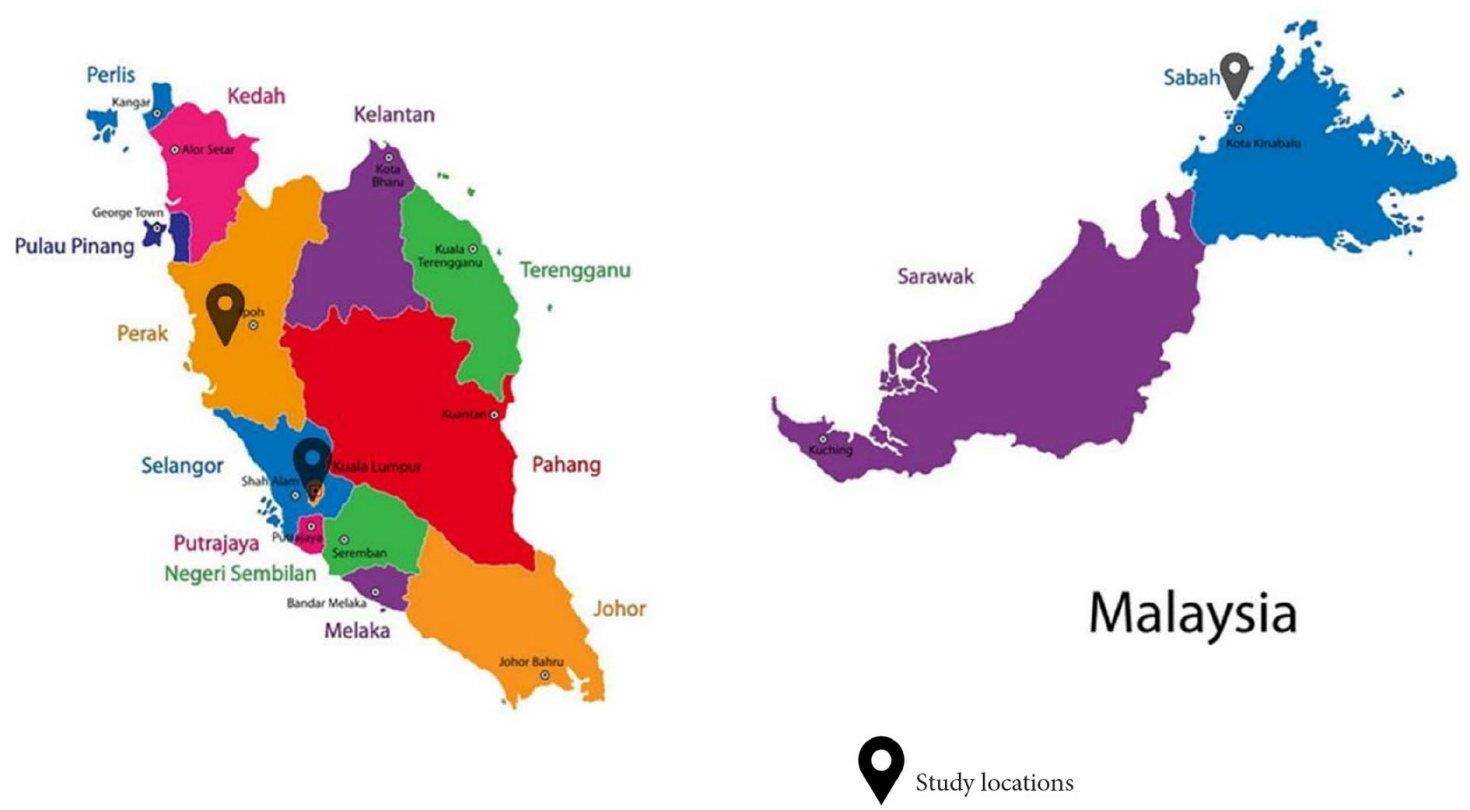
Map of study locations.

This study aimed to investigate the impacts of climate change on children in Malaysia, with a special focus on marginalized children in its community-survey component. It is the first exploratory study in Malaysia with children as the focus of actions aimed at mitigating climate change and environmental degradation. This paper describes the methods and major findings of the research project, which consists of two main components: an environmental epidemiology study (on climate, air pollution and haze) and community surveys conducted in three geographically different locations in Malaysia (i.e., Pulau Gaya, Sabah; Pos Kuala Mu, Perak; and Kuala Lumpur; Figure 1).

## Method

### Specific health sector climate indices from regional climate model downscaling

This study provides physical evidence of climate change in Malaysia over the last two decades.

To this end, using a dynamic downscaling technique (14, 15), we refitted a regional climate model with data from two regions of Malaysia: Kuala Lumpur and Pulau Gaya, Sabah.

#### Study area

The closest available meteorological station for Pulau Gaya is the Kota Kinabalu Airport station, which is about 5 km away from the study area. For Kuala Lumpur, the data from the meteorological station at Subang Airport was used. The Malaysian Meteorological Department maintains both stations.

#### Data analysis

Data was sent to the World Meteorological Organization's Global Telecommunication System and made available at the NOAA's National Climatic Data Center (NCDC) website (https://data.noaa.gov/dataset/dataset/global-surface-summary-of-the-day-gsod) as the global surface summary of the day data sets (GSOD) (16). Data was available in daily resolution. In the current study, the variables obtained and used included the maximum temperature (TX), minimum temperature (TN) and precipitation (PREC). For the Kota Kinabalu Airport station, data was available from 1976 to 2019—spanning 44 years—while the data from Subang Airport station started in 1997. Projection simulations based on Representative Concentration Pathways (RCPs) (i.e., RCP45 and RCP8.5) from a subset of the CORDEX-SEA experiment were used to assess future climate changes. Data was available at a 25 km × 25 km grid resolution and downscaled from CMIP5 GCMs. Downscaled data from two GCMs (i.e., CNRM-CM5 and HadGEM2-ES) using the RCM RCA4 were used. Details on the CORDEX-SEA simulation experiment can be found in Tangang et al. (17) and Malaysia (14).

In the current study, the focus of the assessment was based on health sector-specific climate indices as depicted in Table 1. These indices are identified by the Expert Team on Sector-Specific Climate Indices (ET-SCI). For each year, the climate indices defined in Table 1 are computed separately to form the annual time series. These selected indices are considered proxies of the relevant hazards associated with climate extremes relevant to public health sectors and were computed from the CORDEX-SEA simulated daily meteorological variables:

a) The maximum near-surface air temperature;
b) The minimum near-surface air temperature; and
c) The precipitation of the grids (25 km × 25 km) containing the Kota Kinabalu Airport station.

**Table 1:** Health sector-related climate indices examined.

**Indices**	**Description**
HWN	Heat wave number. A heat wave is defined as three or more days where T*X* > 90th percentile of *TX*
HWD	Heat wave Duration. The length of the longest heat wave identified by HWN
HWM	The mean temperature of all heat waves identified by HWN
CDD	Consecutive dry days. The maximum dry spell length in a year
CWD	Consecutive wet days. The maximum wet spell length in a year

Future changes in these indices were calculated as the relative changes between the future values and the historical (1986–2005) reference values from the regional climate model's output. The future periods are divided into three 30-year epochs. The early twenty-first-century span a period from 2011 to 2040, the mid-twenty-first century from 2041 to 2070 and the end of twenty-first century from 2071 to 2100.

### Association between air pollution and childhood respiratory admissions: A time-series analysis in Sarawak and Kuala Lumpur, Malaysia

The second environmental epidemiological study estimates the risk of childhood respiratory diseases attributable to ambient air pollution in Asia using time-series analyses. This study was conducted in two Malaysian urban areas (Klang Valley and Kuching). The two areas were chosen due to their high levels of ambient air pollution due to urbanization and transboundary haze.

#### Study area

Klang Valley is a Malaysian urban area centered around Kuala Lumpur and encompassing cities and towns in Selangor and Putrajaya. Klang Valley is the most populous city in Malaysia and the country's industrial and commercial hub, with ~8 million inhabitants. Meanwhile, Kuching is Sarawak's (a state in East Malaysia) capital and the most populous city. The city is located at the southwest tip of Borneo and has a population of ~325,000 people. Kuching's main industrial sectors are agriculture and forestry. This study included children below 18 years of age from Klang Valley and Kuching. It is assumed in this study that sick children were transported immediately from their homes to the nearest hospitals.

#### Exposure assessment

We used data provided by the Department of Environment (DOE) Malaysia's Continuous Ambient Air Quality Monitoring Station (CAAQMS) on the daily ambient air pollution data, including measurements of average 24-h PM_10_, SO_2_, NO_2_, CO and O_3_ concentrations between 1 January 2010 and 31 December 2018. Data on children aged 0–17 years hospitalized for respiratory diseases (ICD10: J00-J99) were obtained from two primary sources: the Malaysian Health Data Warehouse (MyHDW), derived from all Ministry of Health (MOH) hospitals in Malaysia, and an electronic database of Hospital Canselor Tuanku Mizan, a teaching hospital in Kuala Lumpur. A total of 17 hospitals in Klang Valley and Sarawak were included. Demographic information (including age and gender) was also obtained from the MyHDW.

#### Data analysis

Poisson regression in a single- and multiple-pollutant generalized linear model (GLM) with a log function was used in this study to estimate the relationship between pollutants and hospital admissions on various lag days. These models were used to regress the dependent variable—the daily number of hospital admissions—on the independent variables (pollutant concentrations). The covariates were the time variable (day), the daily mean temperature, the relative humidity, a holiday indicator and the day of the week. The Poisson models incorporated natural cubic spline functions to account for long-term seasonality patterns in daily hospital admissions as well as other time-varying covariates that could confound the relationship between air pollution and hospital admission. The procedure began with developing the basic core model, which was then adjusted for seasonality, trends and potential confounders. The basic core model was constructed separately for each group (total hospital admissions, boys, girls, under 5 years, 5–9 years and 10–17 years). The quasi-Akaike information criterion (qAIC) was used during the GLM application to determine the number of degrees of freedom (df) for a time in the core model. Based on previous studies, a natural cubic spline of 3 df per year was used for the temporal trends, temperatures and humidity for all groups (18, 19). The Poisson regression estimated the excess risk (ER) effects of a 10-unit (10 μg/m^3^) increase in air pollutants over a 7-day lag period. The ER per 10 μg/m^3^ increment of each pollutant (except CO, where the ER was per 1 mg/m^3^) was calculated as ER = (RR – 1) × 100. This study used a simple GLM regression over a 7-day lag period. All statistical analyses were performed using the R Studio version 1.2.5033 with the “mgcv” and “splines” packages (20).

### Case-crossover study: Assessing the health effects of wildfire haze among children in Malaysia

The third study applied a case-crossover design to examine the association between haze and under-five mortality in Malaysia (21). The study highlighted the need to consider different aspects of haze exposure assessments, namely duration, intensity and time lags. This adds to the limited number of epidemiological studies that have conducted exposure assessments of haze *via* binary or categorical variables that are derived from particulate matter (PM) concentrations (22), the Pollutant Standards Index (23), visibility (24), intensity (25) and duration (25, 26).

#### Study area and data sources

This study included 12 districts in Malaysia during the time period of 2014–2016. The study areas were selected based on the population size (>500,000) and the availability of monitoring stations. The 12 districts that fulfilled the criteria were Kuala Muda, Timur Laut, Kinta, Kuala Lumpur, Klang, Petaling, Melaka Tengah, Johor Bahru, Seremban, Kuantan, Kota Bharu and Kuching. The data for under-five mortality were provided by the Family Health Development Division, MOH Malaysia. Only mortality from natural causes (ICD-10: A00-R99) was included in the analysis part of this study. Using a bootstrap with 10,000 simulations, the empirical under-five mortality rate was 8.2 (95% CI 7.9, 8.5) per 1,000 live births per year.

The environmental data (air pollutants and weather) were provided by the Air Quality Division, DOE Malaysia. For air pollutant data, the data from the monitoring station in the district was used. As weather data was available at all monitoring stations, the temperature and relative humidity (RH) data was averaged from the monitoring stations over the whole state of each district.

#### Exposure assessment

Binary indicators of haze days were derived *via* a combination of duration and intensity. The intensity was determined by the PM10 concentration. Four levels of intensity were considered: (i) Intensity-1 (PM10 > 50 μg/m^3^), (ii) Intensity-2 (PM10 > 75 μg/m^3^), (iii) Intensity-3 (PM10 > 100 μg/m^3^) and (iv) Intensity-4 (PM10 > 150 μg/m^3^). These intensity levels were based on several air quality standards for PM10 (21), including the WHO Air Quality Guidelines (50 μg/m^3^), Malaysia New Ambient Air Quality Standards (AAQS) of 2020 (100 μg/m^3^) and the Malaysia AAQS during the study period (2015) (150 μg/m^3^). Additionally, an intermediate level (75 μg/m^3^) between the Malaysia New AAQS and WHO guidelines was included. Besides the intensity criterion, three durations were considered based on the number of successive days with PM10 > 50 μg/m^3^: (i) Duration-1 (PM10 > 50 μg/m^3^ for 1 day), (ii) Duration-2 (PM10 > 50 μg/m^3^ for two consecutive days) and (iii) Duration-3 (PM10 > 50 μg/m^3^ for > three consecutive days). For each haze binary indicator defined by the combination of intensity and duration, lagged effects of up to 7 days were examined.

#### Data analysis

A time-stratified case-crossover method was applied to explore the association between haze and under-five mortality. This method allows for adjustments for seasonal patterns and long-term trends through its design (27). A generalized additive regression model with a Poisson distribution was applied, adjusting for temperature and RH in the model. Each case was matched with a stratum of days of the week in the same month and year. The districts were treated as random effects in the model. Additionally, indicators of influenza were adjusted for in the sensitivity analysis. The data on influenza in Malaysia were obtained from FluNet (28), the Global Influenza Surveillance and Response System of the WHO. The results of this study were reported as an odds ratio (OR) with a 95% confidence interval (95% CI). All statistical analyses were conducted using the R statistical software (29).

### Community surveys

#### Study locations

The community surveys were conducted in three distinct locations: Pulau Gaya of Sabah (6°1.0798′N 116°1.7909′E); Pos Kuala Mu, Sungai Siput (U), Perak (4°50.2499′N 101°20.0445′E); and People's Housing Project (PPR) Sungai Bonus, Setapak, Kuala Lumpur (3°11.1778′N 101°43.3215′E). The populations in all three locations consist of marginalized communities: documented and undocumented Bajau groups in Pulau Gaya, Sabah; Temiar indigenous people (Orang Asli) in Sungai Siput (U), Perak; and urban poor families in PPR Sungai Bonus, Kuala Lumpur. These three communities live in three distinct geographical settings: an island, a mountainous region and a city at a confluence of the Klang and Gombak Rivers in a vast valley bordered by a few mountain ranges, respectively. These areas were chosen as locations for the community surveys to demonstrate the climate patterns and trends of climate change and environmental degradation as well as the degree of behavioral changes in children living in these particular areas.

#### Study design

A cross-sectional survey was conducted in these communities.

#### Instrument

A set of standardized questionnaires was developed to gather information on related topics in the three study locations (Appendix 1 in Supplementary material). These questionnaires consisted of six sections regarding the respondent's (i) socio-demographical profile; (ii) health status related to diseases, wellbeing and hygiene; (iii) family socio-economical background and living conditions; (iv) accessibility and mobility; (v) perceived impact of climate change and environmental degradation; and (vi) coping mechanism employed due to the climate change and environmental degradation. A six-point Likert scale ranging from 0 = Not Sure, 1 = Strongly Disagree, to 5 = Strongly Agree was used for all questions related to the respondent's perceptions. The questionnaire was pre-tested at the first study location and then adopted at the remaining two locations. Expert reviews were gathered for face and content validity. These experts were given a validation form that contained all the questions for community surveys and were asked to rate items based on their relevancy and clarity using four points: [1 (not relevant), 2 (somewhat relevant), 3 (quite relevant) and 4 (highly relevant)]. Scale-level CVI using Universal Agreement among experts (S-CVI/UA) was calculated. The scale for each item was dichotomised by combining values 3 and 4 together as relevant and values 1 and 2 together as not relevant. In S-CVI/UA, the number of items considered relevant is divided by the total number of items. The S-CVI/UA for relevancy and clarity of the questionnaire was 1.0 (30). As for reliability, Cronbach's alpha value for internal consistency was 0.673, which indicates an acceptable level of reliability (31).

#### Data collection and sample size

The study population was informed by the head of the community and their teachers about the study before the data collection activities. The community surveys were conducted in selected community settings and carried out during different periods from August to December 2020 (Table 2). The sampling method carried out was purposive sampling; however, due to the enforcement of the Movement Control Order by the Malaysian government to curb the COVID-19 pandemic, the number of respondents was restricted. Total respondents for each study location were 200, 196 and 62 in Pulau Gaya, Pos Kuala Mu and PPR Sungai Bonus, respectively.

**Table 2:** Details of participants in the community surveys.

**Location**	**Pulau Gaya, Sabah**	**Pos Kuala Mu, Perak**	**PPR Sungai Bonus, Kuala Lumpur**
	6°1.0798′N116°1.7909′E	4°50.2499′N101°20.0445′E	3°11.1778′N101°43.3215′E
Name of participating schools/community	SK Pulau Gaya (Primary school)	SK Pos Kuala Mu (Primary school)	PPR Sungai Bonus, Kuala Lumpur
	SMK Pulau Gaya (Secondary school)	SMK Bawong (Secondary school)	
	Alternative Learning Centre (ALC), Kampung Lok Urai		
Survey area	SK Pulau Gaya	Kampung Bersah prayer hall (surau)	PPR Sungai Bonus Community Hall
Survey period	6–12 August 2020	15–18 September 2020	26–27 December 2020
Number of respondents	200	196	62

#### Inclusion and exclusion criteria

A self-administered technique with minimum guidance was adopted for questionnaire data collection during the surveys. The inclusion criteria for participants were children aged 6–18 years old, parental consent and being physically healthy. Respondents who were absent during the data collection were excluded from the study.

#### Statistical analysis

Data was entered and analyzed using the IBM SPSS version 23 software. Data cleaning was conducted before analysis to avoid any incorrect or duplicate data that may affect the analysis. Descriptive data using percentage and frequency were used to represent responses for categorical data in the three locations related to respondents' demographics: health status, wellbeing and hygiene, socio-economic and living conditions, and accessibility and mobility to basic amenities. The exploratory factor analysis through the principal component analysis (PCA) method was used on the 41 perceptions statements related to respondents' perceptions to study how the study locations uniquely generate their own indicators. The factor analysis provided correlations between the statements and components—known as factor loadings. Certain statements generated more than one-factor loading—known as cross-loadings, which simultaneously measure more than one component. To overcome this, the factor loading was rotated and redistributed to ensure that each statement measured only one component. Statements with a factor loading of < 0.6 were omitted from the component. The interpretation of the components was addressed as an indicator in this study, and each location generated its own group of indicators with a different set of statements based on the respondents' responses.

## Results

### Specific health sector climate indices from regional climate model downscaling

Annual time series of maximum dry and wet spells identified from the daily rainfall data collected at Kota Kinabalu Airport from 1976 to 2019 showed that the dry spell length over the study area has reduced over the past four decades, while the wet spell length has increased over time. Based on the heatwave definition as per Table 1, heatwave events were not recorded until the early 2000s.

**Figure 2:**
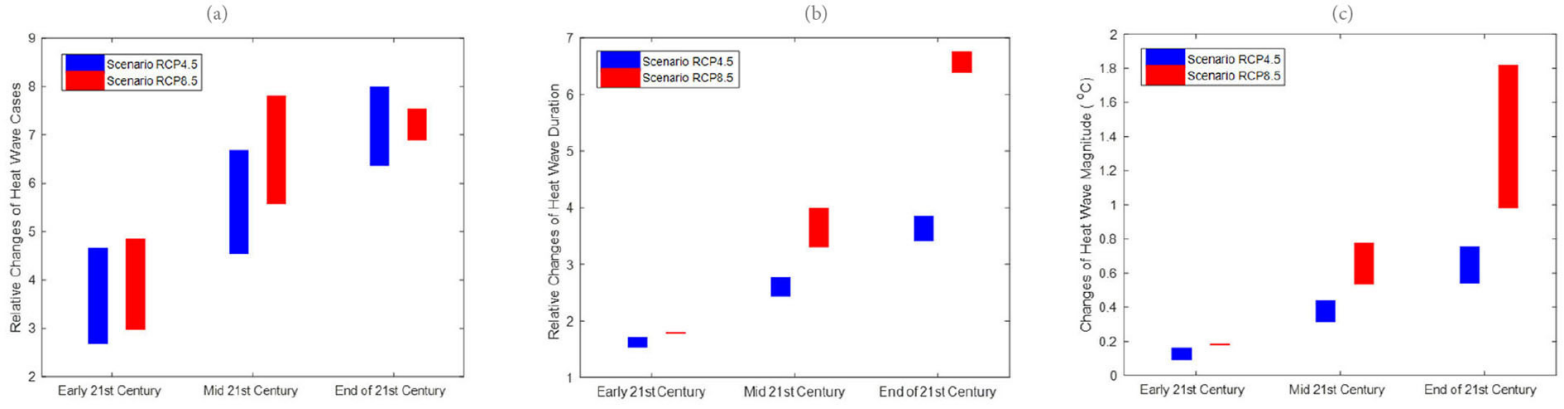
Relative changes of future **(a)** heat wave number, (HWN) **(b)** duration (HWD), and **(c)** magnitude (HWM) for the early 21st century, mid-21st century and end of 21st century for both RCP4.5 and RCP8.5 downscaled projections to historical period (1976–2005) at Kota Kinabalu.

Figure 2 shows the projected changes in terms of heatwave number, duration and magnitude for the three different epochs. The ranges of bars are the projected ranges of the changes. The heatwave number was projected to increase dramatically in the future. The likelihood of heatwave occurrences was projected to be three to seven times higher compared to the historical period. Changes in magnitude could be 1–1.8°C greater than the historical period (Figure 2).

**Figure 3:**
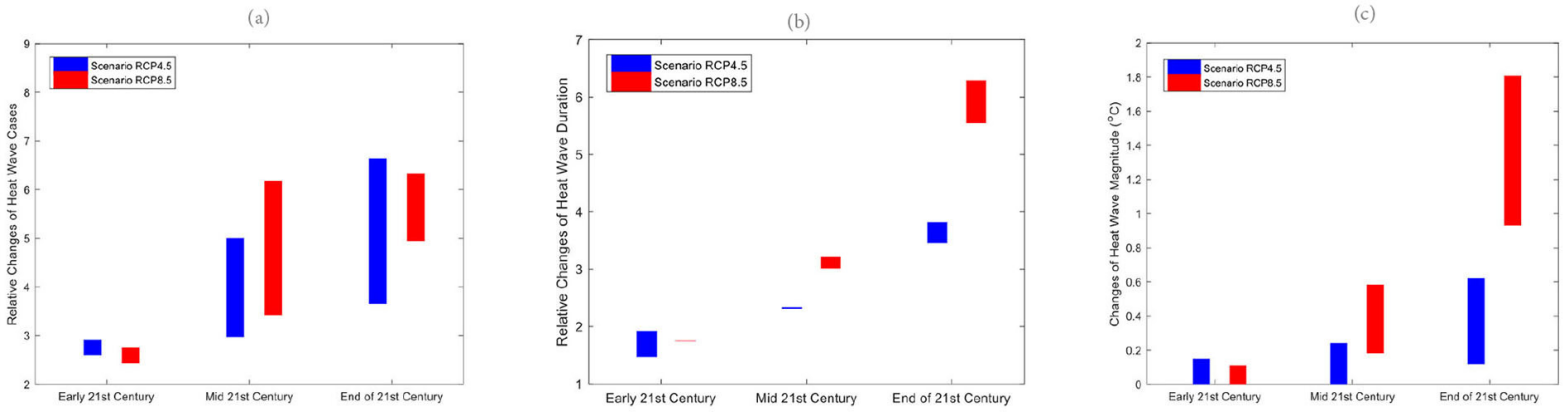
Relative changes of future **(a)** heat wave number (HWN), **(b)** duration (HWD), and **(c)** magnitude (HWM) for the early 21st century, mid-21st century and end of 21st century for both RCP4.5 and RCP8.5 downscaled projections to historical period (1976–2005) at Subang.

**Figure 4:**
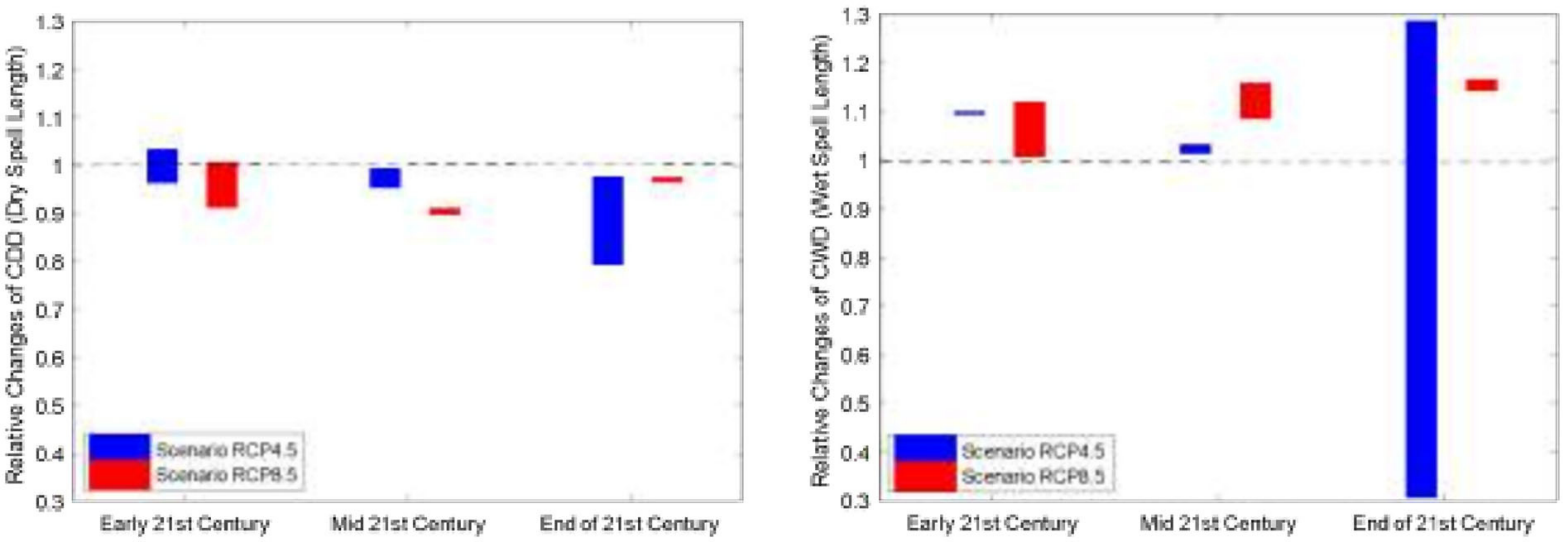
Relative changes of dry spells **(left panel)** and wet spells **(right panel)** for the early 21st century (2011–2040), mid-21st century (2041–2070) and end of 21stcentury (2071–2100) for both RCP4.5 and RCP8.5 downscaled projection at Kota Kinabalu. The dotted line indicates no changes between the future and the historical period.

**Figure 5:**
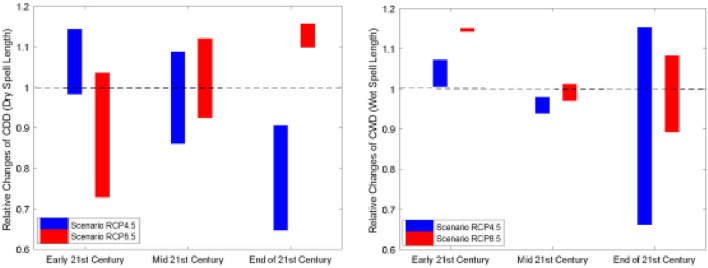
Relative changes of dry spells **(left panel)** and wet spells **(right panel)** for the early 21st century (2011–2040), mid-21st century (2041–2070) and end of 21st century (2071–2100) for both RCP4.5 and RCP8.5 downscaled projection at Subang. The dotted line indicates no changes between the future and the historical period.

Similar to Kota Kinabalu, the heatwave number in Subang was projected to increase dramatically in the future, indicating an increased risk of extreme heating issues in temperature-sensitive socio-economic sectors (Figure 3). Overall, the increasing wet spell length in RCP8.5 suggests the likelihood that wet spells will continue to lengthen in the future (Figure 4). Like Kota Kinabalu, the projection of rainfall-associated indices at Subang is rather noisy and uncertain in the future (Figures 4, 5). As mentioned earlier, rainfall can be a non-linear response to the different degrees of emissions and radiative forcings.

### Association between air pollution and childhood respiratory admissions: A time-series analysis in Sarawak and Kuala Lumpur, Malaysia

**Figure 6:**
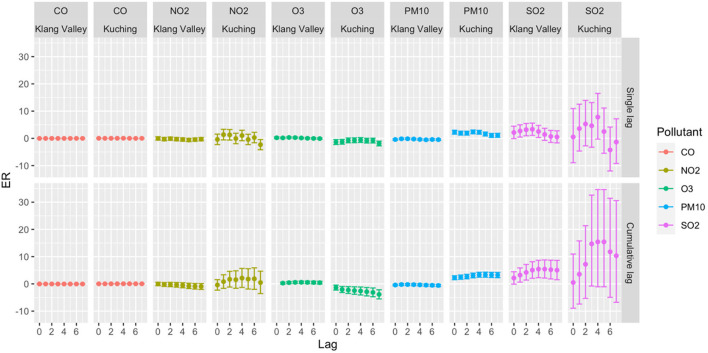
ER with 95% CI of total daily hospital admission with 10 μg/m^3^ increase of pollutant concentrations in Klang Valley and Kuching for 2010–2018.

**Figure 7:**
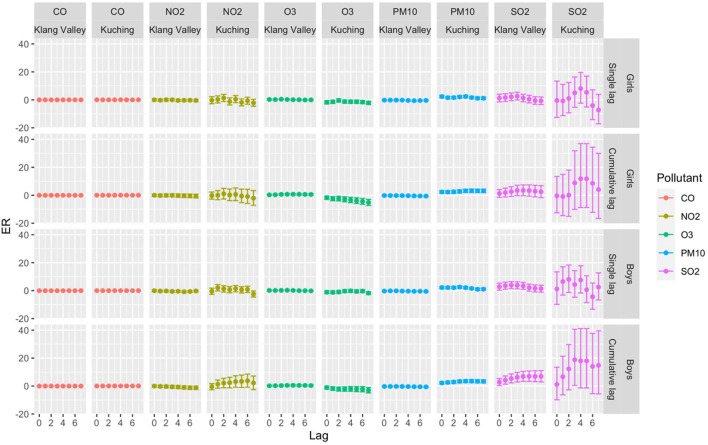
ER with 95% CI of total daily hospital admission by gender with 10 μg/m^3^ increase of pollutant concentrations in Klang Valley and Kuching for 2010–2018.

**Figure 8:**
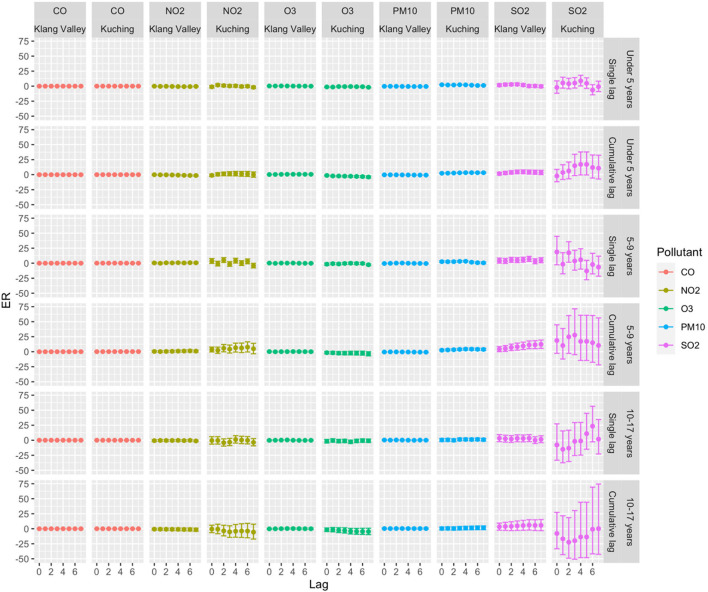
ER with 95% CI of daily hospital admissions by age group with 10 μg/m^3^ increase of pollutant concentrations in Klang Valley and Kuching for 2010–2018.

Table 3 summarizes the descriptive analysis of hospital admissions for respiratory diseases among children and the environmental parameters in Klang Valley and Kuching. From 1 January 2010 to 31 December 2018, Klang Valley had 179,699 hospital admissions for childhood respiratory diseases. Of all the children hospitalized for respiratory diseases, 57.8% were boys and 42.2% were girls. According to the age group, there were 77.7% of children aged 0–4 years, 15.4% of children aged 5–9 years and 6.9% of children aged 10–17 years. Meanwhile, 32,373 children were hospitalized in Kuching for respiratory diseases. In Kuching, of all the children hospitalized for respiratory diseases, 59.9% were boys and 40.1% were girls. About 80.3% of children aged 0–4 years, 14.3% of children aged 5–9 years old and 5.3% of children aged 10–17 years were admitted for respiratory diseases in Kuching. The trends in daily hospital admissions for respiratory diseases in both Klang Valley and Kuching showed an increase in respiratory admissions throughout the study period. Our results shown in Figures 6–8 highlight the excess risk (ER) estimates and 95% confidence intervals associated with an increase in pollutant concentration of 10 μg/m^3^ obtained for each pollutant (only CO the ER was for every 1 mg/m^3^ increment) and evaluated for 7-day lag effects (lag of 0–7 days).

**Table 3:** Summary statistics of daily hospital admissions for respiratory diseases, air pollutants concentration and meteorological factors in Klang Valley and Kuching from 2010 to 2018.

	**Klang Valley**								**Kuching**							
**Characteristic**	**Total**	**Mean**	**SD**	**Min**	**P25**	**Median**	**P75**	**Max**	**Total**	**Mean**	**SD**	**Min**	**P25**	**Median**	**P75**	**Max**
**Respiratory diseases**	179,699	55	23.54	1	37	52	71	131	32,373	10	5.99	0	5	9	14	36
**Gender**																
Boys	103,833	32	14.01	1	21	30	41	79	19,396	6	3.86	0	3	5	8	21
Girls	75,866	24	10.68	0	15	22	30	61	12,977	4	2.89	0	2	3	6	18
**Age group**																
0–4 years	139,560	43	18.80	1	28	40	56	108	26,005	8	5.04	0	4	7	11	33
5–9 years	27,698	9	4.83	0	5	8	11	31	4,641	2	1.43	0	0	1	2	10
10–17 years	12,362	4	2.40	0	2	3	5	14	1,727	1	0.78	0	0	0	1	6
**Air pollutants**
PM_10_ (μg/m^3^)		58.92	29.49	22.43	44.51	52.57	63.43	403.00		44.29	24.39	16.00	33.50	39.00	46.50	356.00
SO_2_ (μg/m^3^)		11.03	3.63	3.68	8.58	10.21	12.67	46.17		4.13	1.63	2.86	2.86	2.86	5.71	14.28
NO_2_ (μg/m^3^)		72.49	14.45	32.25	61.86	71.83	82.09	131.05		28.19	8.81	4.11	20.52	28.73	30.78	65.67
O_3_ (μg/m^3^)		111.05	31.97	10.93	87.73	109.31	132.01	236.28		51.89	18.59	2.19	37.16	48.10	62.31	159.59
CO (mg/m^3^)		1.60	0.44	0.56	1.32	1.56	1.83	6.38		0.82	0.41	0.14	0.56	0.70	0.98	0.59
**Meteorological factors**
Temperature		33.27	1.51	23.74	32.50	33.46	34.28	37.56		33.08	2.05	24.00	31.90	33.25	34.50	38.85
Relative humidity (%)		91.41	3.50	71.86	89.54	91.71	94.00	97.86		95.44	2.79	78.50	94.00	96.00	97.50	100.00

CO, carbon monoxide; Max, maximum; Min, minimum; NO_2_, nitrogen dioxide; O_3_, ozone; P25, 25th percentile; P75, 75th percentile; PM_10_, particles with aerodynamic diameter < 10 μm; PM_2.5_, particles with aerodynamic diameter < 2.5 μm; SD, standard deviation; SO_2_, sulfur dioxide.

#### Klang Valley (Kuala Lumpur)

For the total hospital admissions in Klang Valley, significant single-lag and average cumulative-lag effects of SO_2_ and O_3_ were found (Figure 6). The greatest single-lag effect estimation for SO_2_ was observed at lag 3 with ER 3.33 (95% CI 1.10–5.60), and for O_3_, the highest ER was observed at lag 2 with 0.32 (95% CI 0.05–0.59). The highest ERs for cumulative-lag effects were observed at lags 0–5 with 5.41 (95% CI 2.13–8.79) and lags 0–4 with 0.59 (95% CI 0.14–1.04) for SO_2_ and O_3_, respectively. In the single-lag effect for the boys, the highest ER was observed for SO_2_ at lag 2 with ER 3.91 (95% CI 1.42–6.46). The highest ER for girls was observed for O_3_ at lag 2 with ER 0.48 (95% CI 0.15–0.81). In terms of the cumulative-lag effects for boys, the highest ER was observed at lags 0–5 with 6.97 (95% CI 3.28–10.78) and at lags 0–4 with 0.51 (95% CI 0.01–1.02) for SO_2_ and O_3_, respectively. Meanwhile, among the girls, the highest ER for O_3_ in terms of the cumulative-lag effect was found at lags 0–3 with ER 0.71 (95% CI 0.19–1.24). This study shows that both boys and girls in Klang Valley were susceptible to air pollution.

In terms of age group, statistically significant associations (*p* < 0.05) were found for children aged 0–9 years, but not for those aged 10–17 years. Age was an effect modifier of respiratory hospital admissions, with young children being more susceptible to SO_2_. In terms of single-lag effects, the highest association between pollutants and respiratory hospital admissions for the children aged 0–4 years was observed at lag 3 with ER 2.98 (95% CI 0.70–5.31) and lag 2 with ER 0.36 (95% CI 0.08–0.63) for SO_2_ and O_3_, respectively. SO_2_ at lag 5 had the highest single-effect estimation for children aged 5–9 years with 7.09 (95% CI 2.93–11.42). The highest ER was observed in the cumulative lag of 0–4 days for children aged 0–4 years with 4.65 (95% CI 1.44–7.96) and 0.69 (95% CI 0.22–1.16) for SO_2_ and O_3_, respectively. Meanwhile, for children aged 5–9 years, the highest ER for SO_2_ in the cumulative-lag effect was found at lags 0–7 with 12.32 (95% CI 5.56–19.50).

#### Kuching (Sarawak)

A strong relationship was found between PM_10_ and hospital admissions for respiratory diseases among children in terms of total hospital admissions (Figure 6). Significant single-lag and cumulative-lag effects for PM_10_ were observed at all lags. The greatest single-lag effect estimation for the PM_10_ was found at lag 3 with 2.37 (95% CI 1.67–3.07). The highest ER for cumulative-lag effects was observed at lags 0–5 with 3.37 (95% CI 2.45–4.29). Similar to the findings in Klang Valley, both boys and girls in Kuching were also vulnerable to air pollution (Figure 7). Only PM_10_ was significantly associated with respiratory admission in both genders. The highest ER was observed for PM_10_ at lag 3 in the single-lag effect for the boys with ER 2.55(95% CI 1.74–3.37). PM_10_ had the highest ER for girls at lag 4, with an ER of 2.40. (95% CI 1.50–3.30). The highest ER in cumulative-lag effect for PM_10_ was observed at lags 0–4 with ER 3.50 (95% CI 2.45–4.55) and at lags 0–5 with ER 3.19 (95% CI 1.99–4.40) for boys and girls, respectively.

Statistically significant associations were discovered for the 0–4-year and 5–9-year age groups, but not for the 10–17-year age group (Figure 8). Children under 5 years old were susceptible to PM_10_ and SO_2_. In the single-lag effect, the highest association of PM_10_ for children aged 0–4 was observed at lag 0 (ER 2.31, 95% CI 1.56–3.07), and SO_2_ was observed at lag 4 (ER 8.79, 95% CI 0.24–18.07). In the cumulative-lag effects for children aged 0–4 years, the highest ER (3.33) was only found for PM_10_ at lags 0–5 (95% CI 2.36–4.31). For children aged 5–9 years, the findings showed that the relative magnitude of risk for an association of the pollutants with respiratory hospital admissions followed the descending order of SO_2_, NO_2_, PM_10_ and CO. In the single lag effect, the highest association of each pollutant with hospital admissions was observed for SO_2_ at lag 2 (ER 17.41, 95% CI 1.50–35.81), NO_2_ at lag 4 (ER 5.09, 95% CI 1.109.24). PM_10_ at lag 4 (ER 3.19, 95% CI 1.82–4.58) and CO at lag 4 (ER 0.16, 95% 0.09–0.244).

### Case-crossover study: Assessing the health effects of wildfire haze among children in Malaysia

The study by Phung et al. (21) showed no clear association between haze and under-five mortality. A significant positive association was only observed at Duration-3, and Intensity-2 of the “low” category [OR: 1.210 (95% CI 1.000, 1.464)]. The lag patterns revealed that, with longer durations, a higher OR of under-five mortality occurred at shorter lags; though this variation was minimal. The lag patterns at Intensity-2 and Intensity-3 were similar. Districts with “low” exposure (95th percentile of PM_10_ concentration < 100 mg/m^3^) were sensitive to haze days defined at Intensity-2, whereas those with “high” exposure showed higher ORs at Intensity-3 and Intensity-4. The ORs of under-five mortality were increased at shorter lags under two conditions: (i) with increasing duration and (ii) with increasing intensity (except for Intensity-4). The OR increase with increasing duration was minimal, whereas it was more observable over increasing intensity.

### Community surveys

Table 4 presents the descriptive socio-demographic characteristics of the participants in the three study locations. A majority of the respondents for Pulau Gaya, Pos Kuala Mu and PPR Sungai Bonus were in the 10–14-year-old age group at percentages of 69, 69.4, and 71%, respectively. In Pulau Gaya and Pos Kuala Mu, the majority of the respondents were female (57.5 and 63.3%, respectively), whereas, in PPR Sungai Bonus, the majority were male (53.2%). About 50% of the respondents in Pulau Gaya were currently studying at the Alternative Learning Centre (ALC). Meanwhile, 86.7% of the Pos Kuala Mu respondents were in secondary school, and 51.6% of the respondents in the PPR Sungai Bonus were in primary school during the period of this study.

**Table 4:** Socio-demographic characteristics of participants in the cross-sectional surveys.

	**Pulau Gaya**	**Pos Kuala Mu**	**PPR Sungai Bonus**
	***N* = 200**	***N* = 196**	***N* = 62**
	***n* (%)**	***n* (%)**	***n* (%)**
**Age**			
6–9 years old	35 (17.5)		4 (6.5)
10–14 years old	138 (69.0)	136 (69.4)	44 (71.0)
15–18 years old	27 (13.5)	60 (30.6)	13 (21.0) 1(1.5)
Missing data	0	0	
**Gender**			
Male	85 (42.5)	72 (36.7)	33 (53.2)
Female	115 (57.5)	124 (63.3)	29 (46.8)
**Race/Ethnicity**	Suluk: 21 (10.5)	Temiar: 193(98.5)	Malay: 39 (62.9)
	Bajau: 159 (79.5)	Semai: 2(1.0)	Indian: 21 (33.9)
	Others: 20 (10.0)	Chinese:1(0.5)	Others: 2(3.2)
**Guardian**			
Parents	162 (81.0)	169 (86.2)	49 (79.0)
Mother	17 (8.5)	17 (8.7)	8 (12.9)
Father	2 (1.0)	5 (2.6)	3 (4.8)
Guardian	19 (9.5)	5 (2.6)	2 (3.2)
**Education**			
Primary school	24 (12.0)	26 (13.3)	32 (51.6)
Secondary school	76 (38.0)	170 (86.7)	28 (45.2)
Others (ALC/private school)	100 (50.0)		1 (1.6)
Missing data	0	0	1 (1.6)

The basic amenities available for the respondents as well as their level of accessibility and mobility to the nearest town and health-related information are shown in Table 5. A total of 72% of the respondents from Pulau Gaya did not have access to a treated water supply and relied on seawater instead. Almost all the respondents had access to an electricity supply—either from a private electricity provider or generators. The respondents from Pos Kuala Mu mostly reported using water from the river (70.8%), while 100% of respondents of the PPR reported being supplied with pipe water. In both Pulau Gaya and Pos Kuala Mu, the respondents reported being hindered from going into town (23.5 and 73.5%, respectively) due to weather factors.

**Table 5:** Participants basic amenities, accessibility, mobility to town and health-related information.

**Basic amenities, accessibility, mobility toward nearest town and health-related information**	**Pulau Gaya, Sabah**	**Pos Kuala Mu, Perak**	**PPR Sungai Bonus, Kuala Lumpur**
	***N* = 200**	***N* = 196**	***N* = 62**
	***n* (%)**	***n* (%)**	***n* (%)**
**Water source/supply**			
**Water source for daily use**	Seawater	River	Pipe water supply
Yes	56 (28.0)	139 (70.8)	62 (100)
No	144 (72.0)	57 (29.1)	
**Electricity supply**			
Sabah Electricity Sdn. Bhd. (SESB)	119 (59.5)	NR	NR
Tenaga Nasional Berhad (TNB)	NR	159 (81.1)	62 (100)
Shared electricity/connect from other places	57 (28.5)	30 (15.3)	NR
Own generator	13 (6.5)	4 (2.0)	NR
Shared generator	4 (2.0)	1 (0.6)	NR
No electricity	7 (3.5)	2 (1.0)	NR
**Frequency to town/week**			
1 time	101 (50.5)	86 (43.9)	
2 times	56 (28.0)	38 (19.4)	
3 times	28 (14.0)	11 (5.6)	
More than 3 times	15 (7.5)	6 (3.1)	
Once in a while	0 (0)	1 (0.5)	
Never	0 (0)	11 (5.6)	
Don't know		2 (1.0)	
Missing data	0	41 (20.9)	
**Transportation to town (Multiple respond)**			NR
Boat	200 (100)	NR	
Sampan	15 (7.5)	NR	
Bicycle	3 (2.0)	5 (2.6)	
Motorcycle	1 (0.5)	151 (77.0)	
Car	NR	137 (69.9)	
Other	0 (0)	6 (3.1)	
Missing data	48 (24)	0	
**Factors preventing you from going to town (multiple respond)**			NR
Weather	92 (46.0)	138 (70.4)	
Health	34 (17.0)	109 (55.6)	
Financial	29 (14.5)	67 (34.2)	
Transportation	5 (2.5)	52 (26.5)	
Enforcement	33 (16.5)	20 (10.2)	
Safety	–	50 (25.5)	
Others	29 (14.5)	6 (3.1)	
No hindrance	38 (19.0)	27 (13.8)	

NR, not relevant; Irrelevant to the particular community.

The indicators on the perceived impact of climate change and environmental degradation and their respective factor loadings for Pulau Gaya, Pos Kuala Mu and PPR Sungai Bonus are shown in Tables 6–8. Table 6 lists seven indicators for Pulau Gaya, six indicators for Pos Kuala Mu and five indicators for the PPR—all with factor loadings of 0.6 up to 0.9. The first four major indicators for children living in Pulau Gaya were socializing ability, climate resilience, access to healthcare services and social support, and citizenship. In Pulau Gaya, the children perceived socializing ability (factor loading: 0.755–0.836) as the first indicator of being treated equally in school despite their gender. They also perceived themselves as being healthy during bad weather and as receiving good social support. Many were of the opinion that those without proper state registration documentation would face difficulties in receiving assistance from government facilities. Finally, the children also felt that their house was not the safest place during stormy weather conditions.

**Table 6:** Indicators and items with respective factor loadings for Pulau Gaya, Sabah.

**Indicators**	**Items**	**Factor loading**
Socializing ability	I do not observe gender bias in school	0.836
	I feel comfortable being friends with the opposite genders outside school	0.786
	I feel comfortable being friends with the opposite genders in school	0.755
Climate resilience	During the rainy season, my parents/guardian can go to work	0.795
	During the rainy season, I can go to school.	0.77
	During the hot season, my family's health is not affected	0.760
	During the hot season, my health is not affected	0.742
	During the rainy season, my health is not affected	0.643
	My house is the safest place during bad weather	−0.619
Access to healthcare services and social support	It is easy to get medical treatment	0.844
	I have access to health facilities	0.774
	My family has an alternative source of income	0.66
	I feel that disabled people have difficulty to access health facilities	0.660
	I have access to health facilities other than government-owned facilities	0.659
	My family has financial savings	0.641
Citizenship	I feel that undocumented people have difficulty to be accepted into the government school	0.747
	I feel that undocumented people have difficulty to access health facilities	0.804
Healthy Lifestyle	I drink enough water to quench my thirst	0.658
	I wash my hand regularly	0.651
Food Security	My family has an alternative food source	0.663
	My family has adequate food supply	0.625
Education and literacy	My parents/guardians know how to read, write and count	0.709
	I can go to school	0.706

**Table 7:** Indicators and items with respective factor loadings for Pos Kuala Mu, Perak.

**Indicators**	**Items**	**Factor loading**
Healthy lifestyle	I keep myself clean regularly	0.681
	I drink enough water to quench my thirst	0.638
	I throw rubbish everywhere	0.631
	My house is the safest place during bad weather	0.625
Climate resilience	During the rainy season, I can go to school	0.833
	During the hot season, clean water supply is not affected	0.768
	I stay at a nearby building during storms	0.755
	During the rainy season, it's easy to go back to the village	0.716
	During the hot season, my family's health is not affected	0.709
	During the rainy season, my parents/guardians can go to work	0.695
	During the hot season, fire incidence impact is not serious	0.674
	During the hot season, my health is not affected	0.664
	During the rainy season, I'm not worried about my house's safety	0.652
Citizenship	I feel that undocumented people have difficulty to access health facilities	0.731
	I feel that undocumented people have difficulty to be accepted into the government school	0.707
Socializing ability	Being a girl, I'm doing a lot of house chores compared to my siblings	0.65
	I can be friends with people from the opposite gender	0.633
Education and literacy	I can go to school	0.629
		
Access to healthcare services and social support	I have access to health facilities other than the government-owned facilities	0.753
	My family have an adequate food supply	0.751
	I get vaccinated to prevent illness	0.703
	My family has financial savings	0.687
	I work to help my parents/guardians	0.645
	I feel that my house is wide and comfortable	0.624

**Table 8:** Indicators and items with respective factor loadings for PPR Sg Bonus, Kuala Lumpur.

**Indicators**	**Items**	**Factor loadings**
Climate resilience	During the hot season, my health is not affected	0.858
	My house is the safest place during bad weather	0.818
	During the rainy season, my health is not affected	0.798
	During the rainy season, it's easy to go back to my house area	0.708
	During the rainy season, my parents can go to work	0.681
	During the rainy season, its easy-to-get food supply	0.670
	I feel that disabled people have difficulty to access health facilities	−0.790
Healthcare and Health facility	My family encourages me to eat healthily all the time	0.836
	I have access to government health facilities other than the government-owned facilities	0.811
	My family has an alternative source of income	0.707
	I have access to the government health facilities	0.683
	I feel more confident after getting vaccinated	0.604
Social support	My house is wide and comfortable	0.851
	I feel comfortable to be friends with people	0.757
	My family has financial savings	0.716
	In my village, there is a place to seek help during natural disasters	0.646
	My parents/ guardians know how to read, write and count	0.638
Healthy lifestyle	I clean myself regularly to prevent illness	0.805
	I throw rubbish everywhere	0.69
	I get vaccinated to prevent illness.	0.663
Socializing Ability	I do not observe gender bias in school	0.785
	I feel comfortable being friends with the opposite gender in school	−0.739

**Figure 9:**
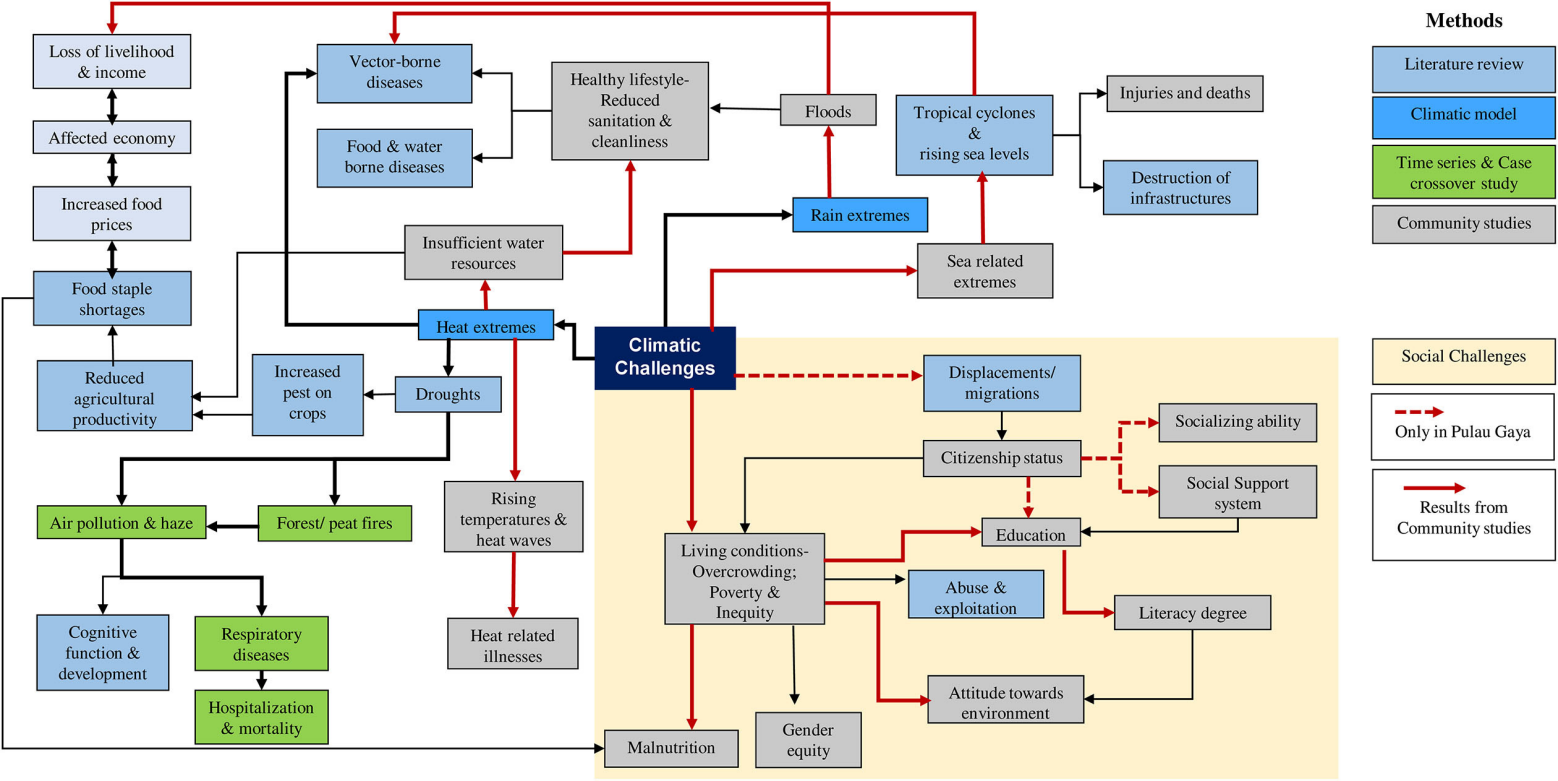
Model of the impacts of climate change on children in Malaysia.

On the other hand, the main indicator for the children's perception of the impact of climate change and environmental degradation based on factor loading for Pos Kuala Mu was climate resilience (factor loading: 0.652–0.833) concerning rain and mobility. During the rainy season, children reported experiencing difficulties in getting to school. On the other hand, they reported little trouble due to heat, since they live at higher altitudes which are much cooler. Another indicator for Pos Kuala Mu was having a healthy lifestyle (factor loading: 0.652–0.681), with personal hygiene perceived as the most important for the children. Similarly, the main indicator for PPR Sungai Bonus is climate resilience (factor loading: 0.670–0.858). There was also a common perception among these students in that they felt uncomfortable in their respective schools—although socializing at school was simultaneously considered a non-issue for them. Based on the results from the climate model, time-series, case-crossover and case studies, we constructed a model showing the short-term and long-term impacts of climatic changes on children (Figure 9).

## Discussion

### Specific health sector climate indices from regional climate model downscaling

As reported in studies from other parts of the world, heat waves have become more frequent in recent decades (32–34). Health-related climatic indices include wet and dry spell lengths as well as heatwave occurrence, duration and magnitude (35). The downscaled projection of future climates based on a subset of CORDEX-SEA (17, 36) simulation outputs suggests a high likelihood of a continual increment of heatwaves in terms of heatwave number, duration and magnitude. The hottest and longest heatwave event ever recorded occurred in 2016 in Southeast Asia during a period of extremely high temperatures in conjunction with the strongest El Niño event extending from 2015 to 2016 (37). In 2016, most of the Southeast Asia Maritime region experienced record-breaking extreme heat events over a period of ~10 days. During this period, the El Nino event contributed to ~49% of global warming; anthropogenic global warming has contributed 29% (38). El Ni1o events have been associated with various disease outbreaks worldwide (39). In Malaysia, El Niño has been associated with heatwave events, haze pollution and related diseases, including the association between heat-related variables and under-five mortality (40) together with cause-specific mortality (41).

In the present study, we report that the heatwave occurrence frequency appeared to be less sensitive to the greenhouse concentration pathways. However, both the duration and magnitude showed considerable dependence on the greenhouse concentration pathways. Duration and magnitude appeared more severe in the higher emissions scenarios. A higher emission scenario (i.e., RCP8.5) is expected to double the duration and magnitude of the heatwave compared to RCP4.5 toward the end of the twenty-first century. On the other hand, rain spell length is projected to increase under the higher emissions scenarios, while future conditions remain very uncertain under the lower emissions scenario (RCP4.5). Dry spell length is projected to become shorter under both the RCP4.5 and RCP8.5 scenarios. Nevertheless, it should be noted that rainfall projections had greater uncertainties and noise levels as compared to the temperature (42). As a corollary, the increased likelihood of extreme heat events under a future warmer climate is also expected to increase the disease burden. Nevertheless, proper quantification of such a specific relationship is challenging due to the nature of impacts from the environmental and climate variabilities that is multifactorial.

### Association between air pollution and childhood respiratory admissions: A time-series analysis in Sarawak and Kuala Lumpur, Malaysia

We found that short-term exposure to ambient air pollution increased the risk of the respiratory disease up to 8 days (7-day lag) after exposure. In the Klang Valley, SO_2_ and O_3_ were significantly associated with increased respiratory hospital admissions, whereas in Kuching, only PM_10_ was significantly associated with increased total admissions for respiratory diseases among children. In Klang Valley, SO_2_ was the most significantly associated pollutant with respiratory hospital admissions, while PM_10_ was the most significantly associated pollutant in Kuching. This observation was expected due to the urban and dense population of Klang Valley, which would lead to higher levels of SO_2_, NO_2_, CO and O_3_ when compared to Kuching. The high level of PM_10_ in Kuching is caused by the burning of local biomass and exacerbated by the (almost) annual transboundary haze from Kalimantan, Indonesia (43).

Most of the related research in Asian countries has found SO_2_ to be linked to an increased number of emergency and outpatient visits for respiratory diseases. In China, every 10 μg/m^3^ increase in SO_2_ concentration was found to be associated with an increase in hospital visits for URTI, pneumonia, and upper and lower respiratory tract infections, with ERs of 2.92 [95% CI 1.88–3.97; (44)], 5.00 (95% CI 1.30–8.80) (45) and 15.17 (95% CI 11.29–19.19) (46), respectively. A similar finding was discovered in an Iranian modeling study, which found a significant association between SO_2_ and acute respiratory diseases at an attributed proportion of 3.65 (95% CI 1.30–5.94) (47).

This study adds to the existing evidence on air pollution and respiratory diseases in children, showing that, despite low levels of SO_2_ (lower than MAAQS) in Klang Valley, the risk of respiratory hospital admissions with the highest ER for a single-lag effect was 3.33 (95 % CI 1.10–5.60) at lag 3 and the highest ER for a cumulative lag-effect was 5.41 (95 % CI 2.13–8.79) at lag 0–5. The source of SO_2_ in Klang Valley is most likely industrial activities (48, 49) and power generation (50) from the power plant located in the city. PM_10_ posed a greater risk to children in Kuching. The lag pattern showed a positive association between PM10 and child respiratory hospitalization, and the effects were higher at shorter lags. This study's findings have strengthened the evidence regarding air pollution and its health effects in Malaysia. Previous case-crossover studies have discovered that respiratory mortality was significantly associated with haze events (PM_10_ > 10 μg/m^3^) for all ages at lag 0 (OR 1.19, 95% CI 1.02–1.40) (22). The findings of this study provide evidence that short-term exposure to ambient air pollution increases the risk of hospitalization for respiratory disease in children. Child age was also identified as a moderator of respiratory hospital admissions, with children aged 5–9 years being more vulnerable to ambient air pollution. Compared to the other age group of children, this group (5–9 years) was more susceptible to air pollution because their lungs are in a period of rapid development and they are active and engage in many outdoor activities (going to school, playing on the playground and participating in outdoor sports) (51, 52).

Furthermore, this study found no gender-based difference in the effects of air pollution on respiratory hospitalisations, which contradicts recent evidence that girls are at a higher risk of respiratory hospitalisations attributable to air pollution than boys (18, 53, 54). Further studies are needed to clarify the differential effects of gender on the association between air pollution and respiratory hospitalization.

### Case-crossover study: Assessing the health effects of wildfire haze among children in Malaysia

This study demonstrated the need to consider duration, intensity and lag in exposure assessments of haze. The reporting of results through a combination of these three aspects would provide more in-depth information to help understand pathophysiological pathways and ultimately contribute to a better risk communication strategy for policy decision-makers and the public. Specifically, lag patterns suggested potentially more acute health risks if haze occurs over longer durations or higher intensities. Policies on preventive measures should consider these aspects for more efficient implementation. Moreover, this study added to the limited understanding of haze health effects in Southeast Asia. The health effects of haze in this region may differ from those reported in other regions (55) due to differences in climate, vegetation and causes of wildfires. Finally, this study contributed to the literature on haze-related health effects in children, especially younger children aged below 5 years old. Children make up a population quite vulnerable to air pollutants, with potentially differentiated health effects in younger vs. older children due to differences in their immune systems (56) and behavior (57).

### Community surveys

From the results, we examined the impact of climate change on children's health in Malaysia based on environmental epidemiology, supported by primary data from community studies that employed a socio-ecological approach. As many researchers have been pointing out, global changes in temperature over time may have a disproportionate impact on children's wellbeing (58–60). For instance, they are more susceptible to diarrheal diseases, skin and eye infections, leptospirosis and vector-borne diseases (e.g., dengue) during the rainy season. In the dry season, health risks to children can include fever and cough, with heatwaves representing a particularly prominent danger in this regard.

The study demonstrated that children from the surveyed locations display degrees of resilience toward climate change and/or environmental degradation—although each location faced specific challenges based on local issues. Specifically, children of Pulau Gaya as well as Pos Kuala Mu dealt with unique challenges during the rainy season, especially in terms of getting to school. According to the IPCC (61), excessive rains are one of the most noticeable events for climate change, leading to unfavorable sea conditions in Pulau Gaya and flash floods in Pos Kuala Mu. Similar phenomena have occurred in Bangladesh, also causing thousands of children to be cut off from their schools (62). However, this was not a problem in PPR Sungai Bonus; these respondents reported their health being impacted during both the rainy and hot seasons instead. Here, the children also perceived that their health was adversely affected during climate extremes. However, since they have access to healthcare and health facilities, social support and realized the importance of a healthy lifestyle, this was not a major concern for them. Heat extremes were also highlighted in our community studies as a source of heat-related illnesses among children that hinders their school attendance. Children in the poor urban community reported similar experiences, most likely due to urban heat islands. The indigenous Temiar children and children in Pulau Gaya also mentioned insufficient processed water as contributing to poor sanitation and self-hygiene. Interestingly, vector-borne diseases would also become another problem, as climate conditions affect the survival and reproductive rates of mosquitoes which, in turn, influences the distribution, abundance, intensity and annual temporal patterns of mosquito activity, particularly their biting rates (63, 64).

Rainy weather extremes also contribute to floods, as demonstrated by our community studies, with floods affecting the family's economy in marginalized communities. The available food supply was also reported to diminish during floods, leading to increases in fresh food demand and prices and thus, adding additional pressure to these communities. Children from affected families often choose to skip school to support their families. In Pulau Gaya, Sabah, we also found families displaced indirectly by climatic challenges (denoted by the dashed red line in Figure 9). This minority group faces difficulties due to their citizenship status, which further impacts the chance of local education for their children and limits their social support system. In this community, children attended NGO-run schools instead, where they are taught basic literacy and numeracy.

Increasing public awareness regarding the importance of education, health, hygiene and environmental management will contribute to these children's wellbeing, building their resilience against climate change and the impacts of environmental degradation. Moreover, certain socio-demographic factors can be strengthened with sufficient knowledge, resources and support to enhance children's adaptive capacity in the face of climate change. A robust adaptation plan and sufficient implementation may help them prepare against impending climactic impacts.

## Conclusion and recommendations

In summary, climate change, environmental degradation and pollution are intensifying in Malaysia alongside the country's rapid development. Accelerated climate change, environmental degradation and pollution present serious risks for children in Malaysia in general. In particular, children living in marginalized communities are even more vulnerable to climate and environmental risks. Based on the results from the studies on specific health sector climate indices, time series, case-crossover and community surveys, we constructed an integrated model that consolidates our overall research processes and demonstrates the crucial interconnections between environmental challenges and impacts exacerbated by climate change (Figure 9). Based on the main findings of this study, several recommendations can be made:

1. Strengthening the Malaysian education sector toward being climate-smart, including enhancing environmental education by focussing on three areas: (i) climate-smart educational content; (ii) climate-smart schools; and (iii) support for teachers with the latest knowledge on climate change science and local impacts and solutions (65).
2. Enhancing and supporting advocacy and representation for children and vulnerable groups.
3. Further research and development to ensure that all child-sensitive climate and environmental governance initiatives are evidence-based.

Although representing only preliminary findings on the status of children in Malaysia, together, the evidence in this study underscores the importance of addressing children's issues in relation to climate change and environmental degradation specifically and not as part of the general population. It is time to re-examine current policies, action plans and international documents on national commitments to climate change and environmental degradation in the context of children. It is hoped that these findings and recommendations will be widely disseminated within relevant networks for further action to be taken at both the community and policymaking levels through effective intersectoral strategies and implementation synergies across all relevant stakeholders.

## Data availability statement

The original contributions presented in the study are included in the article/Supplementary material, further inquiries can be directed to the corresponding author.

## Ethics statement

The studies involving human participants were reviewed and approved by Resaerch Ethics Committee, Faculty of Medicine, National University of Malaysia (JEP-2020-668). Written informed consent to participate in this study was provided by the participants' legal guardian/next of kin.

## Author contributions

MS, IN, SCK, and MM: conceptualization. MS, RD, and YR: validation. MS, HO, and YR: supervision. MS, HO, SCK, and YR: project administration. MS and IN: funding acquisition. MS, HO, RH, MM, IN, RD, and HV: writing—review and editing. SCK, LJ, MFI, VLHP, SNHM, NK, ZIIZ, MM, and LCC: methodology. KC, LJ, MFI, VLHP, SNHM, NK, and ZIIZ: formal analysis. SCK, LJ, MFI, VLHP, SNHM, NK, ZIIZ, LCC, SSZ, MIAW, and NFAB: investigation. SCK, LJ, MFI, VLHP, SNHM, NK, LCC, SSZ, MIAW, and ZIIZ: data curation. SCK and YR: writing—original draft. SCK, YR, and RD: visualization. LJ, MFI, VLHP, SNHM, NK, SSZ, MIAW, NFAB, LCC, and ZIIZ: writing. NFAB: data acquisition and financial management. YR: editing. All authors contributed to the article and approved the submitted version.

## Funding

This research was funded by UNICEF Malaysia (Award No: MLY/PCA202027/PD202021) and co-funded by Universiti Kebangsaaan Malaysia (UKM-NN-2020-041) for the project Analysis of Impacts of Climate Change and Environmental Degradation on Children in Malaysia and Assessment of Child Sensitivity of Current Adaptation and Mitigation Policies.

## Conflict of interest

The authors declare that the research was conducted in the absence of any commercial or financial relationships that could be construed as a potential conflict of interest. The reviewer AN declared a shared affiliation with the author HV to the handling editor at the time of review.

## Publisher's note

All claims expressed in this article are solely those of the authors and do not necessarily represent those of their affiliated organizations, or those of the publisher, the editors and the reviewers. Any product that may be evaluated in this article, or claim that may be made by its manufacturer, is not guaranteed or endorsed by the publisher.
