# Supplementary material for: Impacts of climate change and environmental degradation on children in Malaysia
Source: Front Public Health. 2022 Oct 14;10:909779. doi: 10.3389/fpubh.2022.909779 (PMC9614245; doi:10.3389/fpubh.2022.909779)
Supplement: Supplementary file 1 [file Data_Sheet_1.docx]

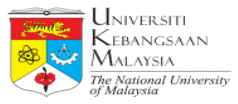

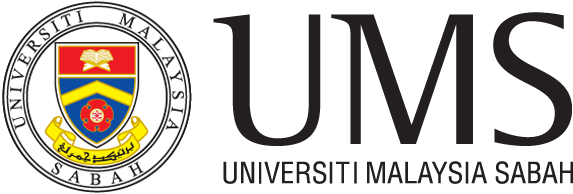


**ANALYSIS OF IMPACTS OF CLIMATE CHANGE AND ENVIRONMENTAL DEGRADATION ON CHILDREN AND IDENTIFICATION OF CHILD-CENTRED CLIMATE ACTION PRIORITIES IN MALAYSIA: A CASE STUDY FOR THE MARGINALISED CHILDREN IN SABAH**

**Salam sejahtera.**

Tuan/Puan yang dihormati :

1. Terlebih dahulu terima kasih kerana memberikan kerjasama dan komitmen dalam penyelidikan ini.
2. Kajian ini bertujuan untuk mendapatkan maklum balas tentang **kesan perubahan iklim dan degradasi alam sekitar terhadap kanak-kanak.** .
3. Segala maklumat yang diberikan dalam borang soal selidik ini hanya untuk kegunaan penyelidikan saja.
4. Anda dikehendaki menjawab kesemua soalan berdasarkan arahan yang dinyatakan di setiap bahagian.
5. Kerjasama anda dalam meluangkan masa memberi maklum balas dengan ikhlas dan jujur adalah amat dihargai dan didahului dengan ucapan ribuan terima kasih.

No. Siri : ______________________

Tarikh : ______________________

Masa : ______________________

Lokasi : ______________________

Jenis Sekolah : ALC (MKN) / Kerajaan / Tidak Bersekolah

Jenis Responden : Normal / OKU

Nama Penyelidik : ______________________

**BAHAGIAN A - PROFIL DEMOGRAFIK RESPONDEN**

***Arahan****:* Sila **tandakan (√)** dan jawab soalan pada ruang yang disediakan

| **A1.** | **Tempat lahir** |  | Dalam Negara |  | Luar Negara |
| --- | --- | --- | --- | --- | --- |

| **A2.** | **Tempat tinggal sekarang** |  |
| --- | --- | --- |

| **A3.** | **Tinggal dengan siapa** |  | Ibubapa |  | Penjaga |
| --- | --- | --- | --- | --- | --- |
|  |  |  | Ibu |  | Bapa |

| **A4.** | **Umur** |  |
| --- | --- | --- |

| **A5.** | **Agama** |  | Islam |  |  | Kristian |  |
| --- | --- | --- | --- | --- | --- | --- | --- |
|  |  |  | Lain-lain (Nyatakan) : ____________________ | | | | |

| **A6.** | **Bangsa/ Etnik** |  | Suluk |  |  | Bajau : ____________________ (Perincikan) | |
| --- | --- | --- | --- | --- | --- | --- | --- |
|  |  |  | Lain-lain (Nyatakan) : ____________________ | | | |  |

| **A7.** | **Jantina** |  | Lelaki |  |  | Perempuan |
| --- | --- | --- | --- | --- | --- | --- |

| **A9.** | **Adakah anda Orang kelainan upaya (OKU)** | | | |  |
| --- | --- | --- | --- | --- | --- |
|  |  | Ya, Nyatakan jenis kelainan upaya : _______________________ |  | Tidak | |

**BAHAGIAN B - KESIHATAN DIRI**

***Arahan****:* Sila **tandakan (√)** dan jawab soalan pada ruang yang disediakan

| **B1.** | **Adakah persekitaran rumah anda tercemar/kotor ?** | | | | |  |
| --- | --- | --- | --- | --- | --- | --- |
|  |  | Ya |  |  | Tidak (Terus ke Soalan B5) | |

| **B2.** | **Pernahkah anda dan/atau ahli keluarga anda mendapat maklumat kesihatan?** | | | | | |
| --- | --- | --- | --- | --- | --- | --- |
|  |  | Ya |  |  | Tidak |  |

| **B3.** | **Dari manakah anda mendapatkan informasi kesihatan ?**  ***(Boleh tanda lebih dari satu)*** | |
| --- | --- | --- |
|  |  | Ibubapa / Penjaga |
|  |  | Televisyen |
|  |  | Radio |
|  |  | Ceramah kesihatan. Nyatakan : ____________________________ |
|  |  | Bertanya terus kepada pegawai kesihatan |
|  |  | Lain-lain. Nyatakan : _____________________________ |
|  |  | Tiada sumber |

| **B4.** | **Apakah maklumat kesihatan yang dikongsikan oleh ibu/bapa/penjaga/ahli keluarga kepada anda? *(Boleh tanda lebih dari satu)*** | |
| --- | --- | --- |
|  |  | Perlu kerap basuh tangan |
|  |  | Perlu menggosok gigi 2 kali sehari |
|  |  | Kurangkan pengambilan makanan atau minuman manis |
|  |  | Pastikan makan makanan yang bersih sahaja |
|  |  | Lain-lain. Nyatakan : _____________________________ |
|  |  | Tiada |

| **B5.** | **Sila Jawab ke semua soalan ini.** | | |  |  |  |  |
| --- | --- | --- | --- | --- | --- | --- | --- |
|  | **a. Penampilan kendiri:** | | |  |  |  |  |
|  | i. Memotong kuku |  | kali seminggu | | | |  |
|  | ii. Menggosok gigi |  | kali sehari | | | |  |
|  | iii. Mencuci rambut dengan syampu |  | kali sehari | | | |  |
|  | iv. Masalah Kutu di rambut |  | Ada | |  | Tiada | |

**b. Masalah kesihatan umum:**

|  | **BIL.** | **MASALAH KESIHATAN** | **YA / TIDAK**  **(√/ X)** |
| --- | --- | --- | --- |
|  |  | Kudis |  |
|  |  | Cirit-birit |  |
|  |  | Alahan (makanan) |  |
|  |  | Selsema |  |
|  |  | Gatal-gatal kulit |  |
|  |  | Sesak nafas |  |
|  |  | Batuk/ kahak berdarah |  |
|  |  | Strok haba |  |

**c. Masalah kesihatan berdasarkan musim:**

|  | Tidak Pernah |  |  |
| --- | --- | --- | --- |
|  | Musim Panas |  | Nyatakan: |
|  | Musim Hujan |  | Nyatakan: |

**BAHAGIAN C - LATAR BELAKANG SOSIO-EKONOMI / LIVING CONDITION**

***Arahan****:* Sila **tandakan (√)** dan jawab soalan pada ruang yang disediakan

| **C1.** | **Pekerjaan ibu/bapa/penjaga :** perlu dikategorikan (the main breadwinner- men) |
| --- | --- |

| **C2.** | **Anggaran Pendapatan Isi Rumah** |  |
| --- | --- | --- |

| **C3.** | **Sumber air untuk kegunaan:** | | |
| --- | --- | --- | --- |
|  | Memasak | Nyatakan: air sungai, air paip, air hujan, air beli |  |
|  | Membasuh | Nyatakan: __________________________________________ |  |
|  | Mandi/Kegunaan lain | Nyatakan: __________________________________________ |  |

| **C4.** | **Apakah anda menggunakan air laut . air sungai/ air paip untuk kegunaan harian ?** | | | | | |
| --- | --- | --- | --- | --- | --- | --- |
|  |  | Ya |  |  | Tidak |  |

| **C5.** | **Sumber bekalan elektrik di rumah** |  | Bekalan elektrik terus dari SESB |
| --- | --- | --- | --- |
|  |  |  | Bekalan elektrik sambungan (dari jiran) |
|  |  |  | Bekalan elektrik ‘generator’sendiri |
|  |  |  | Bekalan elektrik ‘generator’kongsi |
|  |  |  | Tiada |

| **C6.** | **Bilangan isi rumah : _______ ____________** |
| --- | --- |

| **C7.** | **Bilangan tanggungan ibubapa/Penjaga** | Kanak-kanak |  |  |
| --- | --- | --- | --- | --- |
|  |  | Dewasa |  |  |

| **C8.** | **Jenis rumah yang didiami** |  | Rumah kayu atas air |
| --- | --- | --- | --- |
|  |  |  | Rumah kayu atas tanah |
|  |  |  | Rumah konkrit atas air (tiang sahaja) |
|  |  |  | Rumah konkrit atas tanah |
|  |  |  | Lain-lain jenis-jenis rumah. Nyatakan : _______________ |

| **C9.** | **Pemilikan rumah** |  | Rumah sendiri |
| --- | --- | --- | --- |
|  |  |  | Rumah sewa |
|  |  |  | Rumah majikan |
|  |  |  | Lain-lain. Nyatakan : rumah makcik, nenek etc_____ |

| **C10.** | **Bilangan bilik dalam rumah** |  | Tiada bilik |
| --- | --- | --- | --- |
|  |  |  | 1 bilik |
|  |  |  | 2 bilik |
|  |  |  | Lebih dari 2 bilik |

| **C11.** | **Aset yang dimiliki** |  | Tanah |
| --- | --- | --- | --- |
|  | ***(Boleh tanda lebih dari satu)*** |  | Rumah |
|  |  |  | Bot dengan enjin |
|  |  |  | Sampan |
|  |  |  | Peralatan menangkap ikan |
|  |  |  | Lain-lain. Nyatakan : barang antik, motorsikal___ |

| **C12.** | **Peralatan elektrik dalam rumah** |  | Televisyen |
| --- | --- | --- | --- |
|  | ***(Boleh tanda lebih dari satu)*** |  | Set karaoke |
|  |  |  | Peti sejuk |
|  |  |  | Mesin basuh |
|  |  |  | Radio |
|  |  |  | Lain-lain. Nyatakan : astro, kipas, hp, keetle, ricecooker, oven,seterika |

| **C13.** | **Kekerapan makan makanan ruji dalam sehari** | | | | | | | | |
| --- | --- | --- | --- | --- | --- | --- | --- | --- | --- |
|  |  | 1 Kali |  | 2 Kali |  | 3 Kali |  | Lain-lain. Nyatakan: _______ |  |

| **C14.** | **Jenis tandas** |  | Tandas pam dalam rumah |
| --- | --- | --- | --- |
|  |  |  | Tandas pam luar rumah |
|  |  |  | Tandas curah dalam rumah |
|  |  |  | Tandas curah luar rumah |

| **C15.** | **Jenis pengangkutan utama untuk pergerakan di pulau** |  | Bot |
| --- | --- | --- | --- |
|  | ***(Boleh tanda lebih dari satu)*** |  | Sampan |
|  |  |  | Basikal |
|  |  |  | Motosikal |

**SOALAN 14 – 21 NYATAKAN TAHAP PERSETUJUAN ANDA**

***Arahan****:* Jawab soalan dengan tandakan bulat pada skala yang berkenaan.

*(Skala: 0= Tidak Pasti 1= Sangat tidak setuju, 2= Tidak setuju, 3= Kurang Setuju, 4= Setuju, 5=Sangat setuju)*

| **No.** | **Pernyataan** | **Skala** | | | | | |
| --- | --- | --- | --- | --- | --- | --- | --- |
|  |  | 0 | 1 | 2 | 3 | 4 | 5 |
| **C15.** | Saya dapat makanan yang mencukupi setiap hari | 0 | 1 | 2 | 3 | 4 | 5 |
| **C16.** | Rumah saya adalah tempat perlindungan paling selamat semasa cuaca buruk (ribut) | 0 | 1 | 2 | 3 | 4 | 5 |
| **C17.** | Ibu/bapa/penjaga saya tahu membaca, menulis dan mengira | 0 | 1 | 2 | 3 | 4 | 5 |
| **C18.** | Rumah saya cukup luas dan selesa untuk ahli keluarga | 0 | 1 | 2 | 3 | 4 | 5 |
| **C19.** | Pendapatan ibu/bapa/penjaga saya adalah cukup untuk menyara keluarga | 0 | 1 | 2 | 3 | 4 | 5 |
| **C20.** | Saya memilih untuk membantu ibu/bapa/penjaga bekerja untuk menambah pendapatan keluarga daripada ke sekolah | 0 | 1 | 2 | 3 | 4 | 5 |
| **C21.** | Saya biasa membuang sampah di laut | 0 | 1 | 2 | 3 | 4 | 5 |

**BAHAGIAN D - AKSES DAN MOBILITI / ACCESSIBILITY AND MOBILITY**

***Arahan****:* Sila **tandakan (√)** dan jawab soalan pada ruang yang disediakan

| **D1.** | **Kekerapan ke bandar dalam seminggu** | Kali /Seminggu |
| --- | --- | --- |

| **D2.** | **Pernah terhalang untuk ke bandar** | | | | | |
| --- | --- | --- | --- | --- | --- | --- |
|  |  | Ya |  |  | Tidak |  |

| **D3.** | **Faktor penghalang anda ke bandar**  ***(Boleh tanda lebih dari satu)*** | |  |
| --- | --- | --- | --- |
|  |  | Faktor cuaca. *Nyatakan* : _____________________ | |
|  |  | Faktor kesihatan. *Nyatakan* : _____________________ | |
|  |  | Faktor kewangan. *Nyatakan* : _____________________ | |
|  |  | Faktor pengangkutan. *Nyatakan* : _____________________ | |
|  |  | Faktor penguatkuasaan. *Nyatakan* : _____________________ | |
|  |  | Lain-lain. *Nyatakan* : _____________________ | |
|  |  | Tiada halangan | |

| **D4.** | **Dimanakah anda pergi jika anda sakit ? *(Boleh tanda lebih dari satu)*** | |
| --- | --- | --- |
|  |  | Klinik / Hospital kerajaan |
|  |  | Klinik / Hospital swasta |
|  |  | Tradisional/ubat kampung |
|  |  | Rawat sendiri |
|  |  | Tidak mendapatkan rawatan |
|  |  | Lain-lain. Nyatakan : ________________________________ |

| **D5.** | **Dimana anda dapatkan bekalan ubat untuk rawatan masalah kesihatan** | |
| --- | --- | --- |
|  |  | Klinik kesihatan |
|  |  | Farmasi swasta |
|  |  | Kedai |
|  |  | Penjaja ubat tepi jalan |
|  |  | Lain-lain. Nyatakan : ________________________________ |

**SOALAN 6 – 15 NYATAKAN TAHAP PERSETUJUAN ANDA**

***Arahan****:* Jawab soalan dengan tandakan bulat pada skala yang berkenaan.

*(Skala: 0= Tidak Pasti 1= Sangat tidak setuju, 2= Tidak setuju, 3= Kurang Setuju, 4= Setuju, 5=Sangat setuju)*

| **No.** | **Pernyataan** | **Skala** | | | | | |
| --- | --- | --- | --- | --- | --- | --- | --- |
| **D6.** | Saya mudah untuk dapatkan rawatan kesihatan dari Klinik Desa / Klinik kerajaan | 0 | 1 | 2 | 3 | 4 | 5 |
| **D7.** | Saya mudah untuk hadir ke sekolah | 0 | 1 | 2 | 3 | 4 | 5 |
| **D8.** | Jantina saya tidak menjadi masalah ketika berada di sekolah | 0 | 1 | 2 | 3 | 4 | 5 |
| **D9.** | Saya senang bergaul dengan rakan berlainan jantina di Sekolah | 0 | 1 | 2 | 3 | 4 | 5 |
| **D10.** | Saya lebih banyak buat kerja di rumah daripada adik-beradik lelaki/perempuan saya | 0 | 1 | 2 | 3 | 4 | 5 |
| **D11.** | Saya selesa bergaul dengan rakan lelaki/perempuan di luar kawasan sekolah | 0 | 1 | 2 | 3 | 4 | 5 |
| **D12.** | Keluarga saya mempunyai akses kepada khidmat kesihatan klinik kerajaan | 0 | 1 | 2 | 3 | 4 | 5 |
| **D13.** | Saya merasakan orang tiada dokumen susah diterima masuk ke sekolah | 0 | 1 | 2 | 3 | 4 | 5 |
| **D14.** | Saya merasakan orang tiada dokumen susah mendapatkan kemudahan kesihatan | 0 | 1 | 2 | 3 | 4 | 5 |
| **D15.** | Saya merasakan orang kelainan upaya (OKU) susah mendapatkan kemudahan kesihatan | 0 | 1 | 2 | 3 | 4 | 5 |

**BAHAGIAN E - KESAN PERUBAHAN IKLIM & ALAM SEKITAR**

**SOALAN 1 – 11 NYATAKAN TAHAP PERSETUJUAN ANDA**

***Arahan****:* Jawab soalan dengan tandakan bulat pada skala yang berkenaan.

*(Skala: 0= Tidak Pasti 1= Sangat tidak setuju, 2= Tidak setuju, 3= Kurang Setuju, 4= Setuju, 5=Sangat setuju)*

| **No.** | **Pernyataan** | **Skala** | | | | | |
| --- | --- | --- | --- | --- | --- | --- | --- |
| Pada musim hujan / tengkujuh . . . | | | | | | | |
| **E1.** | Saya dapat ke sekolah | 0 | 1 | 2 | 3 | 4 | 5 |
| **E2.** | Bapa saya dapat turun ke laut | 0 | 1 | 2 | 3 | 4 | 5 |
| **E3.** | Mudah untuk mendapatkan bekalan makanan | 0 | 1 | 2 | 3 | 4 | 5 |
| **E4.** | Kesihatan saya tidak terjejas | 0 | 1 | 2 | 3 | 4 | 5 |
| **E5.** | Pergerakan keluar / masuk Pulau menjadi mudah | 0 | 1 | 2 | 3 | 4 | 5 |
| Semasa air pasang / aras laut meningkat . . . | | | | | | | |
| **E6.** | Saya tidak risau keselamatan rumah saya | 0 | 1 | 2 | 3 | 4 | 5 |
| **E7.** | Bapa saya dapat bekerja | 0 | 1 | 2 | 3 | 4 | 5 |
| Semasa musim panas / kemarau . . . | | | | | | | |
| **E8.** | Kesihatan saya tidak terjejas | 0 | 1 | 2 | 3 | 4 | 5 |
| **E9.** | Kesihatan ahli keluarga tidak terjejas | 0 | 1 | 2 | 3 | 4 | 5 |
| **E10** | Bekalan air bersih tidak terjejas / lebih murah | 0 | 1 | 2 | 3 | 4 | 5 |
| **E11.** | Impak kejadian kebakaran tidak serius | 0 | 1 | 2 | 3 | 4 | 5 |

**BAHAGIAN F - MEKANISME PENYESUAIAN / COPING MECHANISM**

**SOALAN 1 – 12 NYATAKAN TAHAP PERSETUJUAN ANDA**

***Arahan****:* Jawab soalan dengan tandakan bulat pada skala yang berkenaan.

*(Skala: 0= Tidak Pasti 1= Sangat tidak setuju, 2= Tidak setuju, 3= Kurang Setuju, 4= Setuju, 5=Sangat setuju)*

| **No.** | **Pernyataan** | **Skala** | | | | | |
| --- | --- | --- | --- | --- | --- | --- | --- |
| **F1.** | Saya ada akses kepada rawatan kesihatan selain klinik kerajaan | 0 | 1 | 2 | 3 | 4 | 5 |
| **F2.** | Keluarga saya ada sumber kewangan alternatif selain daripada sumber kewangan utama | 0 | 1 | 2 | 3 | 4 | 5 |
| **F3.** | Keluarga saya ada kawasan yang cukup untuk bercucuk tanam bagi tujuan sara-diri | 0 | 1 | 2 | 3 | 4 | 5 |
| **F4.** | Keluarga saya ada sumber makanan lain selain sumber makanan sedia ada | 0 | 1 | 2 | 3 | 4 | 5 |
| **F5.** | Keluarga saya mempunyai simpanan kewangan | 0 | 1 | 2 | 3 | 4 | 5 |
| **F6.** | Di kampung saya ada tempat untuk mendapatkan khidmat bantuan semasa berlaku bencana alam | 0 | 1 | 2 | 3 | 4 | 5 |
| **F7.** | Saya minum air masak secukupnya untuk menghilangkan rasa haus | 0 | 1 | 2 | 3 | 4 | 5 |
| **F8.** | Saya mengelak berada di bot ketika ribut | 0 | 1 | 2 | 3 | 4 | 5 |
| **F9.** | Keluarga saya menggalakkan saya makan makanan berkhasiat semasa musim hujan/tengkujuh | 0 | 1 | 2 | 3 | 4 | 5 |
| **F10.** | Saya kerap mencuci tangan untuk mencegah penularan penyakit | 0 | 1 | 2 | 3 | 4 | 5 |
| **F11.** | Saya mendapatkan suntikan imunisasi untuk mencegah penyakit | 0 | 1 | 2 | 3 | 4 | 5 |
| **F12.** | Saya akan berasa lebih yakin selepas mendapat suntikan imunisasi | 0 | 1 | 2 | 3 | 4 | 5 |

| **A8.** | **Status Kewarganegaraan** |  | Warganegara |
| --- | --- | --- | --- |
|  |  |  | Bukan warganegara dengan dokumen |
|  |  |  | Bukan warganegara tanpa dokumen |
